# L-Cysteine Partially Protects Against Acrylamide-Induced Testicular Toxicity

**DOI:** 10.4274/balkanmedj.2017.0830

**Published:** 2018-07-24

**Authors:** Sedat Kaçar, Varol Şahintürk, Betül Can, Ahmet Musmul

**Affiliations:** 1 Department of Histology and Embryology, Eskişehir Osmangazi University School of Medicine, Eskişehir, Turkey; 2 Department of Medical Biochemistry, Eskişehir Osmangazi University School of Medicine, Eskişehir, Turkey; 3 Department of Biostatistics, Eskişehir Osmangazi University School of Medicine, Eskişehir, Turkey

**Keywords:** Acrylamide, L-cysteine, protective, testicular, toxicity

## Abstract

**Background:**

Acrylamide is a widespread substance with many areas of utilization. Acrylamide also forms a part of high-temperature-processed starchy foods. To date, numerous *in vivo* and *in vitro* studies have documented that acrylamide poses toxic effects on various organ systems.

**Aims:**

To determine the potential protective effect of L-cysteine on acrylamide-induced testicular toxicity.

**Study Design:**

Animal experimentation.

**Methods:**

We randomly divided 28 rats into four groups: control (0.9% saline), L-cysteine (150 mg/kg), acrylamide (40 mg/kg), and acrylamide+L-cysteine. After a 10-day intraperitoneal injection period, we euthanized the animals, recorded their body and testis weights, collected blood samples for serum testosterone measurement, and excised the testes for histopathological and morphometric evaluation. Immunohistochemical scoring of proliferating cell nuclear antigen and bax proteins was performed.

**Results:**

Acrylamide reduced body (p<0.01) and testis weights (p<0.05), seminiferous tubule diameter (p<0.001), and proliferating cell nuclear antigen expression (p<0.05) but increased bax protein expression (p<0.01) and the percentage of seminiferous tubules containing multinucleated giant cells (p<0.001). However, no significant change was observed in serum testosterone level of the experimental groups when compared with that of controls. L-cysteine administered with acrylamide decreased multinucleated giant cell number (p<0.001) and reversed the reduced proliferating cell nuclear antigen positivity (p<0.001) but showed no effect in restoring other parameters compared with the group treated with acrylamide alone.

**Conclusion:**

Considering the dose and duration employed, the present study concluded that L-cysteine partially protects testis against acrylamide-induced toxic effects.

Acrylamide (AA), first produced in Germany in 1853 and used as a commercial product since 1954, is a water-soluble chemical compound. AA is used in polymers, cosmetics, paper and textile industries, wastewater treatment, and other applications (1). AA is also used to produce gels for electrophoresis in research labs. After the discovery of AA in food in 2002, its toxic relevance has become increasingly important (2). Considerable amounts of AA were reportedly found in various products, such as bread, biscuits, crackers, breakfast cereals, French fries, potato chips, and coffee (3). These foods are processed at high temperatures above 120 °C (baking, frying, cooking, and roasting). AA formation is based on Maillard reaction, which occurs between amino acids, mostly asparagine, and reducing sugars such as glucose and fructose (4). AA belongs to group 2A carcinogens, in accordance with The International Agency for Research on Cancer Report, indicating that this compound is probably carcinogenic to humans (5). AA poses neurotoxic, carcinogenic, and genotoxic effects on laboratory animals. AA also reduces the number of sperms and Leydig cells and decreases testosterone level. On the other hand, this compound increases vacuolization, DNA damage, the number of multinucleated giant cells (MNGCs) (6), cell cycle delay (7), and the number of morphologically abnormal sperms and apoptotic cells (8). MNGCs are one of the most specific and common indicators of seminiferous tubule (ST) degeneration in testicular toxicity studies. The number of MNGCs is directly proportional to the degree of toxicity (9). Therefore, examining the existence and quantity of MNGCs in testes is a crucial method used in testicular toxicology studies.

L-cysteine (L-cys) is one of the sulfur-containing amino acids. Other sulfur-containing amino acids include methionine, homocysteine, and taurine, but only L-cys and methionine are joined into proteins. L-cys is a precursor of inorganic sulfates, coenzyme A, and glutathione. L-cys is also a semi-essential amino acid, and it can be synthesized in the mammalian liver. In addition, L-cys contributes to the synthesis of structural proteins, prevents oxidation of nerve cells, maintains the stability and rigidity of connective tissues, and reduces inflammatory reactions by speeding the healing process of the body (10). As an acetylated derivative of L-cys, N-acetyl cysteine (NAC) has entered the World Health Organization’s List of Essential Medicines (11). NAC is an antioxidant that exerts beneficial effects on semen parameters and testis morphology. NAC also promotes glutathione synthesis and blocks oxidative damage as a chelator (12,13). NAC is a xenobiotic, whereas L-cys is a natural substance for the human body. Given this difference, we preferred L-cys over NAC in the present study.

In this study, we investigated the potential protective effects of L-cys on AA-induced testicular toxicity in rats.

## MATERIALS AND METHODS

### Animals

A total of 28 Sprague-Dawley rats (11-12 weeks old and 330±20 g body weight) were obtained from the Local Medical and Surgical Experimental Research Center of the university. The animals were fed with standard pellet food and tap water *ad libitum* in appropriate cages. A total of 3-4 animals were housed per cage at room temperature; appropriate humidity was provided (45%-50%). The rats were exposed to 12 h light and 12 h dark cycles. The study protocol was approved by the Local Ethics Committee for Animal Experimentation of the university (26.04.2014, approval number 406/2014).

The rats were randomly assigned into four groups (n=7): control (0.9% saline solution), L-cys (150 mg/kg/bw), AA (40 mg/kg/bw), and AA+L-cys (40 and 150 mg/kg/bw). L-cys was administered 45 min before AA injection. All injections were administered intraperitoneally for a period of 10 days at 9 am. Dose selection and duration were determined in accordance with previous studies (14,15).

At the end of the experiment, 24 h after the last injection, the rats were anesthetized by xylazine (90 mg/kg/bw)+ketamine (10 mg/kg/bw) and euthanized. Afterward, blood samples were collected into sterile tubes via cardiac puncture, and the testes were excised with a median scrotal incision.

### Chemicals

In the present study, we used the following chemicals: L-cys (Catalog No: C7352, Sigma-Aldrich, St. Louis, MO, USA), AA (Catalog No: A9099, Sigma-Aldrich, St. Louis, MO, USA), mouse/rat testosterone enzyme-linked immunosorbent assay (ELISA) total testosterone kit (Catalog No: RTC001R, BioVendor GmbH, Kassel, Germany), xylazine (Alfazyne, Alfasan IBV, Holland), ketamine (Ketalar^®^, Eczacıbaşı, İstanbul, Turkey), anti-bax (Cat # sc526, Santa Cruz), and anti-proliferating cell nuclear antigen (PCNA) primary antibodies (Cat # CM152B, Biocare Medical).

### Body, testis, and relative testis weight measurements

Body weights were measured at the beginning and end of the experiment, whereas testis weights were recorded at the end of the study. Body weight measurements were performed using a digital kitchen scale with a sensitivity of 1 g (SKS 4507, Sinbo Corporation, China), whereas testis weight measurements were performed using another digital precision scale with a sensitivity of 0.0001 g (Adventurer Pro AV264C, Ohaus Corporation, Pine Brook, NJ, USA). Both scales came with valid calibration certificates. Before testis weight measurement, all adjunct tissues around the testes were carefully removed using a bistoury. Right and left testis weights were summed up for calculation of total testis weight for each rat. Then, the relative testis weight for each rat was calculated using the following formula:

(Total testis weight/Body weight) × 100

### Testosterone measurement

Blood samples were centrifuged at 1000 g for 15 min to separate the serum from blood cells. The serum samples were then stored at -20 °C for subsequent testosterone analysis. Serum total testosterone levels were measured using a mouse/rat testosterone ELISA kit (BioVendor, Czech Republic; Catalog No: RTC001R) in accordance with the manufacturer’s manual. The ELISA testosterone kit, which was used in this experiment, is a competitive immunoassay for quantitative measurement of testosterone in rat serum samples. The principle of the assay is a solid-phase ELISA based on competitive binding. The absorbance of wells was determined by VICTOR^TM^ X3 Multilabel Plate Reader (PerkinElmer, USA) at 450 nm. Testosterone levels of the samples were determined by a standard graph prepared with the absorbance values of standards and expressed as ng/mL. The analytical sensitivity of the rat testosterone kit was 0.066 ng/mL, and intra-assay and inter-assay CV totaled 6.50% and 11.3% respectively. The cross-reactivity of steroid hormones reached as low as <0.1% for testosterone and 69.6% for dihydrotestosterone. The calibration range was 0.1-25 ng/mL, and calibrator concentrations reached 0, 0.1, 0.4, 1.5, 6.0, and 25.0 ng/mL.

### Tissue processing

The testes were fixed in Bouin’s solution. Thereafter, each testis was cross-sectioned into approximately three equal parts and dehydrated in ascending graded ethyl alcohols. After placing into xylol, the cross-sections were embedded in paraffin. Finally, we obtained three separate paraffin blocks (A, B, and C) for each testis. Then, we cut four 3-4 μm thick sections from the A, B, and C blocks with randomly-trimmed distances for staining with hematoxylin-eosin (H-E), bax, PCNA, and H-E again. In this manner, we obtained six sections for H-E, three for bax, and three for PCNA staining. All six sections reserved for H-E were stained. On the other hand, two sections (one from A block and one from C block) were stained for bax and PCNA.

### Microscopic evaluation

Six H-E stained testis sections for each rat were evaluated by two histologists in a blind manner for histopathological alterations in interstitial spaces (edema, vascular congestion, inflammation, and Leydig cell damage) and STs (tubular basal membrane damage, spermatogenetic and Sertoli cell damages, MNGC presence, and tubular lumen changes). Micrographs were obtained using a BX51 light microscope (Olympus Corporation, Tokyo, Japan) attached with a DP70 digital camera (Olympus Corporation, Tokyo, Japan).

### Percentages of seminiferous tubules containing multinucleated giant cells

MNGCs were not observed in the control and L-cys groups. Thus, cell counting was carried out only in AA and AA+L-cys groups. Scoring was conducted according to the existence of MNGCs. If one ST presented at least one MNGC, it was scored as “1” regardless of how many MNGCs existed. A total of 120 different and randomly selected STs were obtained from the six sections of each rat (6×20). Thus, 840 (120×7) different tubules were considered for each group. Then, the percentages of MNGC-containing STs for each rat were calculated with the following formula:

(STs number having at least one MNGC/Total ST number counted) × 100

### Seminiferous tubule diameter measurement

We used an image analyzing software (BS 200Pro Plus Version 3.0, BAB Image Analyzing Systems, Ankara, Turkey) for the measurement of ST diameter (STD). By drawing parallel transverse lines, 50 randomly selected STDs from six sections of each rat were measured. Therefore, 1400 STDs (350×4 groups), or 350 (50×7 rats) per group, were recorded.

### Immunohistochemistry

Antigen retrieval was performed by heating in a microwave oven for 20 min in 10 mM citrate buffer. Then, endogenous hydrogen peroxidases were inactivated with 3% H_2_O_2_ treatment. The sections were blocked with ultra V blocking solution and incubated overnight at 4 °C with diluted primary antibody against PCNA (1:100, monoclonal) or bax (1:100, polyclonal), which were obtained from mice and rabbits, respectively. Thereafter, the sections were incubated with secondary antibodies; (3-amino-9-ethylcarbazole) was used as a substrate. Counterstaining was performed using hematoxylin. Ultimately, four tissue slides (two for bax and two for PCNA) were obtained for each rat.

In this study, a previously reported semi-quantitative scoring system (16) was adapted to score the testis and used by two histologists for double blind evaluation of immunostained testicular sections. Under the light microscope, 7-8 STs in each section were evaluated in three randomly-selected 20X objective areas. A total of 15 STs were determined for each rat. We considered only the spermatogonia and primary spermatocytes because they were mostly cells that react with bax or PCNA. If a STs contained <40% of those cells, it was regarded as negative and if ≥40%, positive. On the other hand, immunostaining intensity was scored as follows; 0: negative, 1: weak, 2: moderate, and 3: strong. Thereafter, percentages of positively stained STs for each rat were calculated and scored as follows: 0%-4%=1, 5%-19%=2, 20%-39%=3, 40%-59%=4, 60%-79%=5, and 80%-100%=6. Finally, histo-, additive, and multiplicative Quick scores were calculated.

### Statistical analysis

Statistical analysis was carried out using SPSS software (Statistics for Windows, Version 21.0. IBM Corp. Armonk, NY, USA). All data were tested for normality using the Shapiro-Wilk test. Normally distributed data were compared with one-way analysis of variance (ANOVA). If a statistically significant difference was found across groups, we performed homogeneity of variance test. When variances were homogenous, post-hoc Tukey’s test was applied. The initial and final body weights, total testis, and relative testis weights were analyzed in the same manner.

The non-parametrical Kruskal-Wallis test was carried out when data were not distributed normally. Then, Dunn’s test was used for pairwise comparisons. Serum testosteron levels, STD, PCNA, and bax scores were tested using such method. For analysis of STs percentages containing MNGC, Mann-Whitney U test was performed as only AA alone and AA+L-cys groups presented MNGCs. The values of p<0.05 were regarded as significant in all statistical analyses. The data followed-up by ANOVA were expressed as mean ± standard deviation, whereas the data followed-up by Kruskal-Wallis or Mann-Whitney U tests were expressed as the median (Q25-Q75) and minimum-maximum values. As performing power analysis is inappropriate for non-significant and non-parametrically-analyzed data, we only calculated the statistical powers of the final body and total testis weights; their values reached 92% and 88%, respectively.

## RESULTS

### Observational results

**Figure 1:**
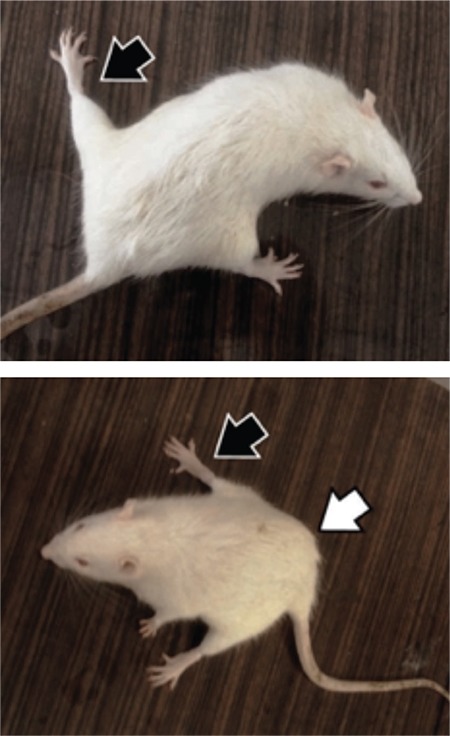
Rats after six-day acrylamide treatment. Note that the hind legs of the rats were splayed, and body posture was disrupted. Black arrows: splayed leg, white arrow: abdomen rubbing to the ground.

The rats in the control and L-cys groups were active and mobile. Rats treated with AA alone reflected the opposite behavior during the two halves of the experiment. In the first half (first 5 days), the rats were extremely aggressive and out of control before injection. On the other hand, in the other half of the experiment (last 5 days), the rats became numb and lethargic and stood still in their cages. The animals exhibited difficulty in using their hind limbs and moving, and their abdominals remained on the ground. Their hind limbs were spread and splayed (Figure 1). The rats also showed poor appetite. The behavior of AA+L-cys-treated rats was not as extreme as that of rats treated with AA alone.

### Body, testis, and relative testis weights

As depicted in Table 1, initial body weights showed no statistical difference among the groups. However, both the final body weight means of AA alone- and AA+L-cys-treated groups manifested significant reduction compared with the control group (p<0.01). AA-treated (p<0.01) and AA+L-cys-treated (p<0.05) groups showed significant weight reduction compared with the L-cys alone group. When the initial and final body weights were compared in each respective group, a significant body weight increase in the L-cys alone group (p<0.05) and a significant body weight decrease in the AA (p<0.001) and AA+L-cys groups (p<0.01) were observed. Total testis weight results showed that AA (p<0.05) and AA+L-cys (p<0.01) groups recorded a significant decrease when compared with both the control and L-cys alone groups, as shown in Table 1. No significant difference was noted among groups with respect to relative testis weight.

### Serum total testosterone

No statistically significant difference was observed with regard to total testosterone levels among groups, as shown in Table 2. However, the testosterone levels were lower in the AA-treated groups when compared with the control and L-cys alone groups. The lowest median values were detected in AA-treated (3.62 ng/mL) and AA+L-cys-treated (0.75 ng/mL) groups. Consistently, the minimum testosterone values were observed in AA-treated (0.48 ng/mL) and AA+L-cys-treated (0.33 ng/mL) groups. The maximal testosterone levels were detected in the control (34.74 ng/mL) and L-cys alone (21.61 ng/mL) groups.

### Microscopic evaluation

**Figure 2:**
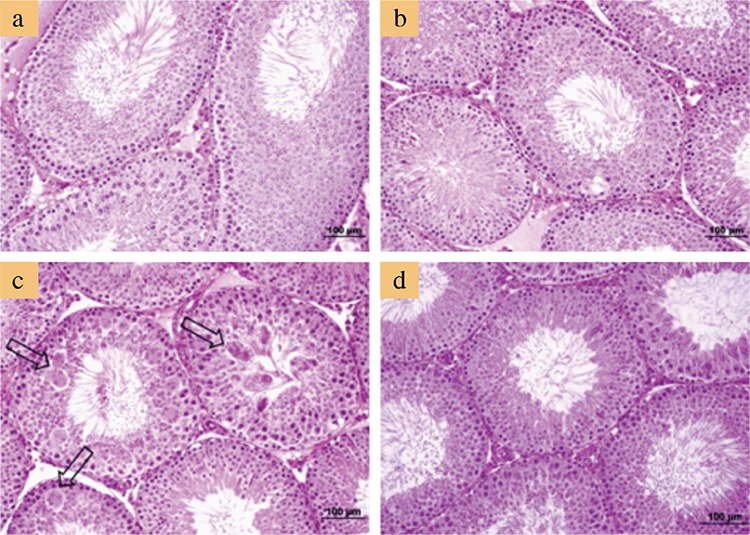
Representative micrographs of testicular sections. Normal histologic appearance in control (a) and L-cysteine groups (b). Seminiferous tubules containing multinucleated giant cells (arrows) in acrylamide group (c). Normal histologic appearance in acrylamide+L-cysteine groups (d). Note that multinucleated giant cells are conspicuous in the acrylamide-alone group. Sections were stained with hematoxylin and eosin. Bars represent 100 μm.

**Figure 3:**
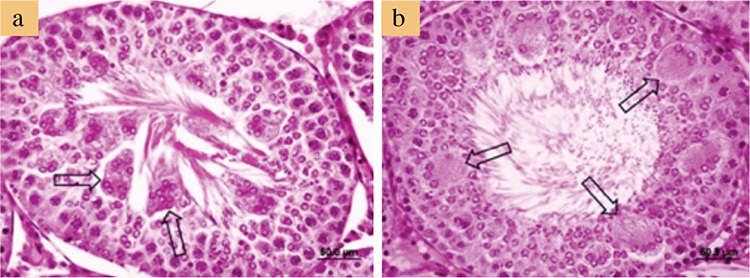
Photomicrographs of acrylamide alone-treated rats. Multinucleated giant cells (arrows) exfoliated into the lumen (a) and were found among spermatogenetic cells (b). Sections were stained with hematoxylin and eosin. Bars represent 50 μm.

Figure 2 and 3 show the representative photomicrographs of all studied groups. Both control and L-cys groups showed normal testicular histology in the STs and interstitial fields (Figure 2a, 2b, respectively). In AA-treated group, numerous MNGCs were observed in the STs, whereas the interstitial fields and other structures appeared normal (Figure 2c, 3a, 3b). Several MNGCs lay close to the STs lumen (Figure 3a), whereas others were found among spermatogenetic cells (Figure 3b). In the AA+L-cys-treated group, MNGCs were significantly reduced (p<0.001) in STs (Table 3), whereas the other structures showed a nearly normal histology, as shown in the control and L-cys groups (Figure 2d).

### Percentage of seminiferous tubules containing multinucleated giant cells

The percentages of STs containing MNGCs in AA and AA+L-cys-treated groups were compared. The data indicated the significantly higher percentage of seminiferous tubules containing MNGCs in the AA-treated group than in the AA+L-cys-treated group (p<0.001), as shown in Table 3.

### Seminiferous tubule diameter

As shown in Table 4, the STD medians of AA alone and AA+L-cys groups were significantly lower than that of the control group (p<0.001). When we compared these two groups with the L-cys alone group, the STD median of AA-treated group was significantly lower than that of the L-cys group (p<0.05), whereas the STD of AA+L-cys group registered no significant difference.

### Immunohistochemistry

**Figure 4:**
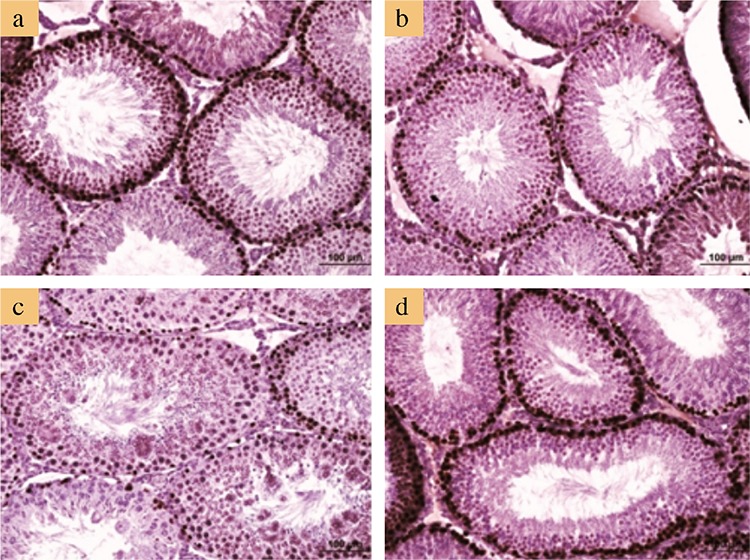
Representative micrographs of proliferating cell nuclear antigen immunostaining. Positive staining notably occurred in spermatogonium and spermatocytes in the control group (a); similar staining was observed in the L-cysteine group (b). Note that proliferating cell nuclear antigen immunostaining decreased notably in seminiferous tubules containing multinucleated giant cells in the acrylamide alone group (c), and it is restored in the acrylamide+L-cysteine group (d). Bars represent 100 μm.

Table 5 presents the semi-quantitative scoring results of PCNA-immunostained testicular sections. According to the results, the median Histo scores of the AA alone group was significantly lower than those of the other three groups (p<0.001), whereas the other groups showed no significant difference from each other. In the STs, predominant primary spermatocytes and spermatogonia were stained as PCNA-positive in the control and L-cys groups. However, PCNA-immunostaining displayed less positivity in the group treated with AA alone when compared with the other groups. Notably, the STs that contained MNGCs displayed less PCNA positivity than the tubules without MNGCs. As for the AA+L-cys group, PCNA positivity was similar to that of the control and L-cys groups (Figure 4a, 4d).

**Figure 5:**
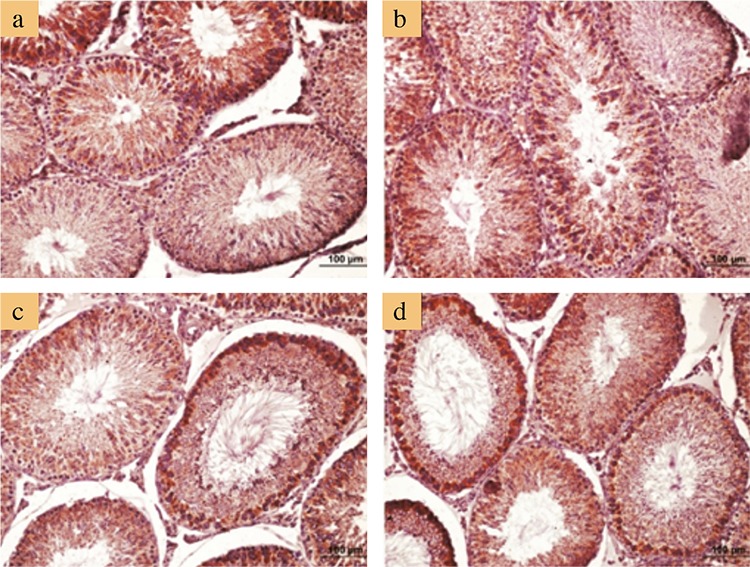
Representative micrographs of bax immunostaining. Normal bax staining in control (a) and L-cysteine (b) groups. Increased bax staining, especially in seminiferous tubules containing multinucleated giant cells in acrylamide alone (c) and acrylamide+L-cysteine (d)-administered groups. Bars represent 100 μm.

Table 6 presents the semi-quantitative scoring results of bax-immunostained testicular sections. According to these results, the median score of the AA-alone group was significantly higher than that of the median scores of the control (p<0.01) and L-cys groups (p<0.05) but exhibited no significant difference from the median score of the AA+L-cys group. With respect to bax-immunostaining, the control and L-cys groups were stained similarly, but both AA-treated groups were stained more positively and exhibited no difference from each other (Figure 5a, 5d).

## DISCUSSION

According to our results, the final body weights showed a significant decrease in both AA-treated groups, consistent with the previous results (17,18). The reason for this decrease may be because AA can reduce the appetite of rats and lead to less food intake (19). Similar to body weight results, testis weights also decreased in both AA-treated groups, as shown in other studies (17,18). Previous studies reported that a significant weight loss in the testis with no notable cell loss occurs due to the reduction of ST fluid (STF) (9). In literature, some inconsistencies exist with respect to relative testis weights, which were reported to increase (19), decrease (20), and remain unchanged (18) following AA administration. Our results showed that no significant difference existed among the relative testis weights of groups. However, relative testis weights of AA-alone and AA+L-cys groups were partially higher than that of the control group. This partial difference might result from two reasons. The first reason might be that AA more seriously affects other organs than testes. The second reason, as suggested in the study of Creasy and Chapin (9), is the notion that the testis preserves its weight much better than any other organ, i.e., any decrease in total body weight results in an increase in relative testis weight. Although L-cys could not significantly alleviate the AA-induced weight loss in the rats, the rats in the AA+L-cys group presented more variable body weights than those in the AA-alone group, indicating that L-cys protected some rats against AA.

As for serum testosterone levels in AA-treated rats, many studies revealed testosterone decreases following AA injection (21,22), whereas some studies reported testosterone increases at certain doses of AA (23). Although we observed a downward trend for serum testosterone levels in AA-treated rats in this study, the groups presented no significant difference. This result can be attributed to the low sample size and marked variation in intra- and inter-groups in our study. We had to keep the sample size low on account of ethical concerns. In addition, the reports mentioned above suggested that AA dose and study duration may affect serum testosterone levels. Specifically, AA increases testosterone level to some extent and then decreases it, indicating that low-dose AA-induced testosterone decrease can be compensated by Leydig cell hyperplasia (18). In the current study, our dosing regimen caused no changes in the testosterone levels among AA-treated groups.

AA decreased STD in our study, and this result is consistent with that of a previous report (24); L-cys could not protect against this decrease. According to Creasy and Chapin (9), two main reasons can explain STD decline: the germ cell loss in STs and the decrease in STF that is released by Sertoli cells. Based on this information, and as no significant germ cell loss was observed in STs, STD declined most probably due to STF decrease in the present study.

AA has been reported to induce MNGC formation in testis (8,14,17,19,22), coinciding with our results. In the present study, we observed a more significant decline in the percentage of STs containing MNGC in the AA+L-cys-treated group when compared with the AA alone group. This significant decline can be explained with either of the following reasons: L-cys as a nucleophile (10) might prevent AA, which is an electrophile, from damaging primary spermatocytes with a specific elimination reaction (25,26), or L-cys, as a precursor of glutathione, might increase the antioxidant capacity of the body.

In addition, we observed several MNGCs in the AA alone group and another few in the AA+L-cys-treated group. In literature related to MNGC formation, Rotter et al. (27) used transgenic mice with MNGCs and explained the formation of MNGCs in these mice in two ways. First, 4N primary spermatocytes fail to undergo complete meiosis and therefore cannot produce haploid sperm cells. Consequently, primary spermatocytes form MNGCs subsequent to partial divisions. The other explanation is the unbroken and extending cytoplasmic bridges between spermatocytes (27).

Notably, we observed MNGCs in two different parts of the ST. Some MNGCs were observed among the spermatogenic cells, especially among primary spermatocytes, whereas other MNGCs were observed near the seminiferous lumen, which showed signs of exfoliation. As mentioned in the study of Rotter et al. (27), MNGCs initially form at the level of primary spermatocytes in the tubules and advance toward the lumen of tubules similar to other spermatogenic cells. However, our experiment only spanned 10 days; this period was too short for exfoliation of MNGCs into the lumen. Therefore, exfoliation of MNGCs into lumen might be attributed to the more rapid occurrence of spermatogenic stages than those of normal spermatogenesis. Another reason why MNGCs exfoliate over a short-time period might be because of the absence of production of secondary spermatocytes and spermatids. The lack of elongated spermatids at points where MNGCs exfoliate into lumen corroborates this postulation.

Consistent with the study of Ma et al. (23), hind-leg splay was observed in the AA-alone and L-cys+AA-treated groups. The latter group included some rats with milder hind-leg splaying. These groups of rats showed an abnormal gait, moved with their abdominal stuck to the floor, and struggled in stepping forward. Tyl and Friedman (28) elucidated this abnormality and stated that AA binds motor proteins kinesin and dynein, thereby halting trans-axonal transport and leading to distal axonopathy.

According to immunohistochemistry results, PCNA expression was diminished by AA treatment and this decrease was prevented by L-cys pre-treatment. AA-evoked PCNA decrease has also been reported in literature (29). Interestingly, L-cys-sustained PCNA expression following AA treatment is likely due to the antioxidant effect of L-cys. For bax protein, both AA-treated groups displayed increased staining. Spermatogonia and spermatocytes were especially more positively stained. The spermatogonia and primary spermatocytes detected in the STs, which contained MNGCs, expressed more bax protein when compared with other cells. AA has been previously reported to increase bax staining (30).

In conclusion and in the light of our findings, AA is suggested to decrease body and testis weights, STD and PCNA expression, and increase MNGCs and bax protein expression but cause no significant change in relative testis index and serum testosterone level. On the other hand, L-cys failed to completely prevent body and testis weight loss, testosterone level, and STD decline induced by AA. However, L-cys protected testis by decreasing MNGCs and preventing AA-induced PCNA decrease. We need further studies to elucidate the exact effects of L-cys on AA-induced testicular toxicity.

## Figures and Tables

**Table 1:**
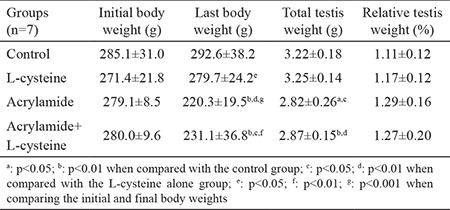
Body, testis, and relative testis weights of groups. Values are expressed as mean ± standard deviation

**Table 2:**
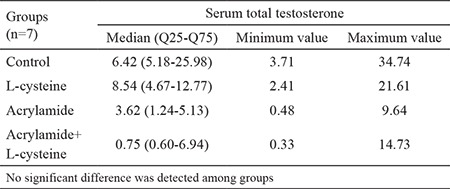
Serum total testosterone levels (ng/mL). Values are expressed as median (Q25-Q75) and minimum-maximum values

**Table 3:**
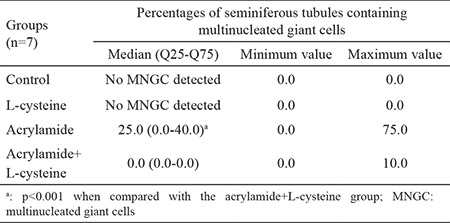
Percentages of seminiferous tubules containing multinucleated giant cells according to the groups. Values are expressed as median (Q25-Q75) and minimum-maximum values

**Table 4:**
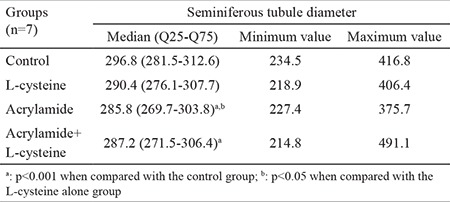
Average seminiferous tubule diameters (μm). Values are expressed as median (Q25-Q75) and minimum-maximum values

**Table 5:**
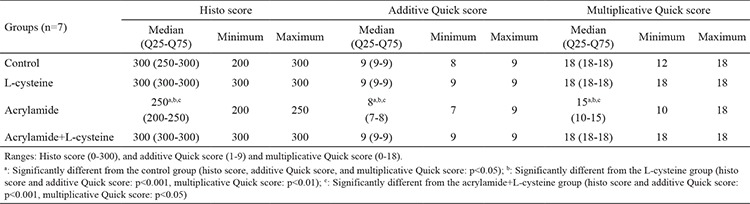
Semi-quantitative scoring results of proliferating cell nuclear antigen-immunostained testicular sections. Data are indicated as median (Q25-Q75) and minimum-maximum values

**Table 6:**
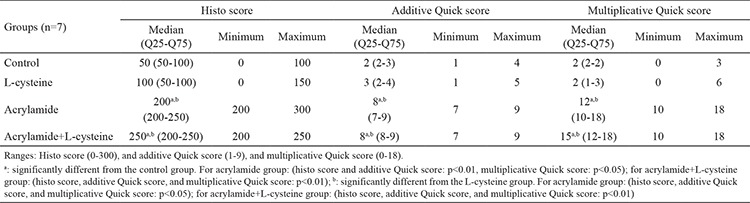
Semi-quantitative scoring results of bax-immunostained testicular sections. Data are indicated as median (Q25-Q75) and minimum-maximum values
